# High-Dose Menaquinone-7 Supplementation Reduces Cardiovascular Calcification in a Murine Model of Extraosseous Calcification

**DOI:** 10.3390/nu7085318

**Published:** 2015-08-18

**Authors:** Daniel Scheiber, Verena Veulemans, Patrick Horn, Martijn L. Chatrou, Sebastian A. Potthoff, Malte Kelm, Leon J. Schurgers, Ralf Westenfeld

**Affiliations:** 1 Division of Cardiology, Pulmonology, and Vascular Medicine, Medical Faculty, University Duesseldorf, Duesseldorf 40225, Germany; E-Mails: daniel.scheiber@med.uni-duesseldorf.de (D.S.); verena.veulemanns@med.uni-duesseldorf.de (V.V.); patrick.horn@med.uni-duesseldorf.de (P.H.); malte.kelm@med.uni-duesseldorf.de (M.K.); 2 Department of Biochemistry, Cardiovascular Research Institute Maastricht, Maastricht University, Maastricht 6229 ER, The Netherlands; E-Mails: m.chatrou@maastrichtuniversity.nl (M.L.C.); l.schurgers@maastrichtuniversity.nl (L.J.S.); 3 Department of Nephrology, University Duesseldorf, Medical Faculty, Duesseldorf 40225, Germany; E-Mail: sebastian.potthoff@med.uni-duesseldorf.de; 4 Cardiovascular Research Institute Duesseldorf, University Duesseldorf, Medical Faculty, Duesseldorf 40225, Germany

**Keywords:** menaquinone-7, vitamin K_2_, cardiovascular calcification, matrix Gla-protein, chronic kidney disease

## Abstract

Cardiovascular calcification is prevalent in the aging population and in patients with chronic kidney disease (CKD) and diabetes mellitus, giving rise to substantial morbidity and mortality. Vitamin K-dependent matrix Gla-protein (MGP) is an important inhibitor of calcification. The aim of this study was to evaluate the impact of high-dose menaquinone-7 (MK-7) supplementation (100 µg/g diet) on the development of extraosseous calcification in a murine model. Calcification was induced by 5/6 nephrectomy combined with high phosphate diet in rats. Sham operated animals served as controls. Animals received high or low MK-7 diets for 12 weeks. We assessed vital parameters, serum chemistry, creatinine clearance, and cardiac function. CKD provoked increased aortic (1.3 fold; *p* < 0.05) and myocardial (2.4 fold; *p* < 0.05) calcification in line with increased alkaline phosphatase levels (2.2 fold; *p* < 0.01). MK-7 supplementation inhibited cardiovascular calcification and decreased aortic alkaline phosphatase tissue concentrations. Furthermore, MK-7 supplementation increased aortic MGP messenger ribonucleic acid (mRNA) expression (10-fold; *p* < 0.05). CKD-induced arterial hypertension with secondary myocardial hypertrophy and increased elastic fiber breaking points in the arterial tunica media did not change with MK-7 supplementation. Our results show that high-dose MK-7 supplementation inhibits the development of cardiovascular calcification. The protective effect of MK-7 may be related to the inhibition of secondary mineralization of damaged vascular structures.

## 1. Introduction

Biological aging [1] and diseases like chronic kidney disease (CKD) [2] or diabetes mellitus [3] are associated with an increased incidence of cardiovascular calcification, an independent cardiovascular risk factor accompanied by enhanced morbidity and mortality [4].

For decades vascular calcification (VC) was thought to be the consequence of passive precipitation of calcium (Ca) and phosphate (P) ions resulting from a supersaturated Ca × P product [5]. Today cardiovascular calcification is appreciated as an actively regulated, cell-mediated process characterized by the interaction of inductive and inhibitory proteins [6]. According to the anatomical localization, VC can be classified as intimal and medial calcification, although a clear-cut distinction is almost impossible in clinical practice [7]. Intimal calcification is linked to atherosclerosis and characterized by inflammatory accumulation of oxidized lipids [8]. Medial calcification develops independently of inflammation and lipid deposition along the elastic fibers. It is a typical consequence of aging and found in patients suffering from chronic kidney disease or diabetes mellitus [9,10,11]. Despite differences in etiology, the underlying pathophysiological mechanism of intimal and medial calcification is similar [6].

The vitamin K-dependent matrix Gla-protein (MGP) is a potent inhibitor of arterial calcification [12]. MGP was presented first in 1983 by Price and colleagues as a 14kD protein purified from bovine bone matrix [13]. That the function of MGP was mainly vascular became clear from MGP-deficient mice, all of which died within a few months of birth due to blood vessel rupture as a result of VC [14]. Likewise, humans with a hereditary dysfunctional MGP (Keutel syndrome) suffer from widely distributed extraosseous calcifications [15]. There are two posttranslational modifications in MGP: gamma-glutamate carboxylation and serine phosphorylation [16]. While the function of posttranslational MGP phosphorylation is not completely understood, Murshed and colleagues showed that MGP has to be carboxylated to prevent VC [17]. Diminished MGP carboxylation is associated with an increased tendency of calcification of the vasculature [18]. Analogous to vitamin K-dependent blood clotting factors (Factors II, VII, IX, X, and Protein C, S, and Z), the biological activity of MGP depends on the presence of vitamin K as cofactor [19]. Current medical treatment with vitamin K antagonists may, besides providing effective anticoagulation, also increase the risk for VC [20].

Patients with CKD are characterized by widespread extraosseous calcification. We as well as others have shown that CKD patients display vitamin K insufficiency, associated with elevated plasma concentrations of inactive MGP [21,22,23]. Increased plasma levels of inactive ucMGP are paralleled by enhanced morbidity and mortality in patients suffering from CKD, aortic stenosis, or congestive heart failure [22,24,25]. Supplementation with vitamin K results in a dose- and time-dependent decrease of ucMGP plasma levels [23,26,27]. So far, no trials reported “hard outcomes” such as VC in the context of CKD and vitamin K supplementation.

Fat-soluble vitamin K is an essential micronutrient [28]. There are two forms of vitamin K in nature: phylloquinone (vitamin K_1_; VK_1_) and the menaquinones (vitamin K_2_; VK_2_; MK-n). VK_1_ is tightly bound to the chloroplast membrane of plants [29]. Menaquinones differ in side chains of varying length. They are described as MK-n, in which n denotes the number of unsaturated isoprenoid residues. MK-7 is produced by bacteria and is present in fermented foods such as cheese or sauerkraut [30]. In this study we used MK-7 because of its long half-life and good bioavailability. Two independent observational studies [31,32] described a protective cardiovascular effect of nutritional intake of menaquinones, whereas no effect was found for phylloquinone. This discrepancy in function was ascribed to the better availability and transport of long-chain menaquinones such as MK-7 as compared to VK_1_. However, in animal models of warfarin-induced vascular calcification, the short chain menaquinone MK-4 was tested, which displayed greater potency in the inhibition of vascular calcification [33,34,35]. The MK-7 supplementation dose of 100 μg/g diet used in this present study was based on these MK-4 studies [33,34,35].

The aim of this study is to evaluate the impact of high-dose MK-7 supplementation on the development of cardiovascular calcification and the impact on cardiovascular function in a murine model of chronic kidney disease characterized by enhanced extraosseous calcification.

## 2. Materials and Methods

### 2.1. Animals and Diets

The animal study protocol was authorized by the responsible governmental office called *LANUV* (“Landesamt für Natur, Umwelt und Verbraucherschutz Nordrhein-Westfalen”; file reference 87-51.04.2010.A275). All experiments were executed according to the German animal welfare act in close cooperation with veterinaries of the Heinrich Heine University. We used 42 male Wistar rats aged 12 weeks with a body weight about 300 g at the beginning of the study protocol. Rats were kept in a climate-controlled room (22–24 °C, relative humidity 60%–80%) with a 12-hour light, 12-hour dark cycle. Food and water were given *ad libitum*.

Animals were divided into an interventional group undergoing 5/6 nephrectomy, receiving a high phosphate diet and into a control group undergoing sham operations. Half of each group received a high MK-7 diet, so that we distinguished between four different treatment groups receiving different diets (sniff-Spezialdiäten GmbH) (Table 1).

Pure synthetic MK-7 was provided by NattoPharma ASA (Hovik, Norway).

**Table 1:** Diets.

Control (Sham-OP)	
Co (*n* = 10*)*	Co-K_2_ (*n* = 10*)*
Standard diet:	MK-7-supplemented standard diet:
0.36% Phosphate	0.36% Phosphate
0.6% Calcium	0.6% Calcium
0.5 µg/g VK_1_	0.5 µg/g VK_1_
0 MK-7	100 µg/g MK-7
**Intervention (5/6-Nephrectomy + high phosphate diet)**	
CKD (*n* = 11*)*	CKD-K_2_ (*n* = 11)
High phosphate diet:	Phosphate- and MK-7-rich diet:
1.2% Phosphate	1.2% Phosphate
0.6% Calcium	0.6% Calcium
0.5 µg/g VK_1_	0.5 µg/g VK_1_
0 MK-7	100 µg/g MK-7

Animals were randomly distributed to four study groups, with two diets different in MK-7 and phosphate concentration. Animals in CKD and CKD-K_2_ groups were 5/6 nephrectomized. Control animals were sham operated. Co = control group; Co-K_2_ = MK-7-supplemented control group; CKD = intervention group; CKD-K_2_ = MK-7-supplemented intervention group; MK-7 = menaquinone-7; VK_1_ = vitamin K_1_.

### 2.2. Study Design

All animals took part in a three-month study protocol. On the first day we measured blood pressure using a tail-cuff system. Blood samples were taken by retro orbital bleeding. Body weight was taken twice weekly. 5/6 nephrectomy was performed according to a surgical technique initially described by Perez-Ruiz [36]. Briefly, we performed right-sided nephrectomy and one week later following recovery from the initial surgery, rats underwent functional 2/3 nephrectomy of the remaining left kidney by careful ligation of the renal parenchyma. Controls underwent a similar two-step laparotomy exposing the kidneys but without nephrectomy. After three and eight weeks we repeated the measurements from the preoperative day. At the end of the study after three months we collected 24-hour urine samples from six animals in each group in metabolic cages. Animals were sacrificed under anesthesia by puncture of the vena cava inferior. Blood was collected for serum analyses. Heart, aorta, and kidney tissues were collected for further analyses.

### 2.3. Echocardiography

Echocardiography was performed as described previously [33]. Briefly, rats were anesthetized using Isoflurane and two-dimensional and M-mode measurements were accomplished using Vivid i, GE Healthcare (GE Healthcare, Buckinghamshire, England) with a 12 MHz probe. Animals were placed in the supine-lateral position with ECGs were obtained throughout the procedure. Parasternal long-axis and short-axis views of the left ventricle (LV) were obtained, ensuring that the mitral and aortic valves and apex were well visualized. Area fraction and wall area were determined by planimetry of end-diastolic and systolic volumes in parasternal short axis. Measurements of LV end-diastolic and end-systolic dimensions were obtained in M-mode at mid-papillary level from more than three beats and fractional shortening (FS) was calculated as FS (%) = ((LVIDd – LVIDs)/LVIDd) × 100, where LVID is LV internal diameter, s is systole, and d is diastole. Diastole is defined as the maximum measurable area; systole is defined as the minimum measurable area. Doppler flow spectrum of the ascending aorta was recorded from the suprasternal view. Peak velocity was measured, and the waveform was also traced to obtain a velocity time-integral calculation and peak gradient.

### 2.4. Blood/Urine Analyses

Blood and urine analyses were performed by Animal Blood Counter (Scil Animal Care Company GmbH, Viernheim, Germany) and in the Institute of Clinical Chemistry of University Hospital Düsseldorf.

### 2.5. Histology

Tissues were perfused with cold phosphate-buffered saline (PBS) solution by cannulation of left ventricle. Afterwards tissues were embedded in TissueTek (Sakura Finetek Europe B.V., Alphen aan den Rijn, the Netherlands) on dry ice for cryofixation, paraffin embedded, or placed in frozen nitrogen. Different histological and immunohistological stainings were performed and analyzed with a Leica DM4000 M RL microscope mounted with a Leica DFC 425C camera (Leica Mikrosysteme GmbH, Wetzlar, Germany). Quantitative measurements were performed with ImageJ software (National Institutes of Health, Bethesda, MD, USA).

### 2.6. qRT-PCR

Real-Time PCR mRNA was extracted using the commercial kits RNAlater and RNeasy (Qiagen, Hilden, Germany) with proteinase K digestion before RNA extraction to maximize mRNA yield. Integrity and amount of mRNA were analyzed by capillary electrophoresis (Agilent Bioanalyzer 2100; Agilent Technologies, Böblingen, Germany). Reverse transcription and real-time PCR were performed with Applied Biosystems 7500 Fast Real Time PCR System (Applied Biosystems, Foster City, CA, USA) according to the manufacturer’s instructions. The expression level in untreated mice was arbitrarily assigned the value 1.0, and all other expression values were expressed as fold changes thereof. Values were analyzed using REST software tool (Quiagen, Hilden, Germany).

### 2.7. Statistics

We performed ANOVA with Bonferroni’s *post hoc* analysis using GraphPad Prism 5 software (GraphPad, San Diego, CA, USA) to estimate the overall differences between experimental groups. Confidence intervals over 95% were regarded as significant.

## 3. Results

### 3.1. Experimental CKD

**Figure 1:**
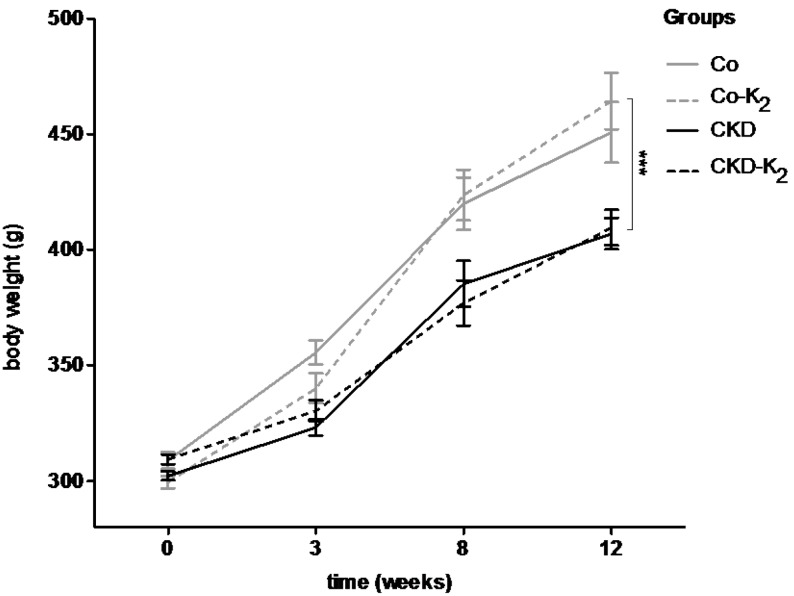
Body weight in grams (g). Body weight of animals was measured repetitively during 12 weeks of study protocol. Experimental animals were not fully grown at start of the experiment; however, all animals gained weight significantly. During the experiment, control animals (Co; Co-K_2_) gained significantly more weight compared to CKD and CKD-K_2_ animals. Co = control group; Co-K_2_ = MK-7 supplemented control group; CKD = 5/6 nephrectomized group; CKD-K_2_ = MK-7 supplemented 5/6 nephrectomized group; g = grams. Significant differences: *** *p* < 0.001.

Combination of surgical 5/6 nephrectomy with supplementation of a high phosphate diet succeeded in mimicking key metabolic features of CKD. Serum creatinine levels were elevated by more than 50% in CKD and CKD-K_2_ animals compared to controls (Table 2) (CKD *vs.* Co after 12 weeks, *p* < 0.001). Likewise, blood urea nitrogen (BUN) concentrations were significantly elevated in the CKD groups (CKD *vs.* Co after 12 weeks, *p* < 0.001). 5/6 nephrectomy and phosphate supplementation affected phosphate concentrations in serum and urine: urine phosphate excretion was more than tripled in CKD animals compared to controls (CKD *vs.* Co after 12 weeks, *p* < 0.001) (Table 2) with an apparent hyperphosphatemia only in CKD but not in CKD-K_2_ rats. There was no difference in hemoglobin or hematocrit concentration between the experimental groups (Table 2). Since severe renal impairment is associated with cachexia, we repetitively assessed body weight. All animals that were not fully grown at the start of the study gained weight during the 12 weeks of the experimental period. Control animals increased significantly more weight compared to CKD and CKD-K_2_ animals (Figure 1) (CKD *vs.* Co after 12 weeks, *p* < 0.01).

**Table 2:** Serum and urine parameters after 12 weeks of study protocol.

Parameters	Co	Co-K_2_	CKD	CKD-K_2_
**Serum creatinine (mg/dl)**				
Week 12	0.3 ± 0	0.3 ± 0	0.5 ± 0	0.5 ± 0
**BUN (mg/dl)**				
Week 12	40 ± 2.2	42 ± 1.3	51 ± 2.9	50 ± 1.9
**Serum phosphate (mmol/l)**				
Week 12	1.8 ± 0.1	1.6 ± 0.1	2.2 ± 0.2	1.8 ± 0.1
**Hb (g/dl)**				
Week 12	15.0 ± 0.5	15.5 ± 0.2	15.5 ± 0.2	15.5 ± 0.1
**Hkt (%)**				
Week 12	45.8 ± 1.3	47.9 ± 0.9	44.0 ± 1.5	45.0 ± 0.3
**Creatinine clearance (ml/min)**			
Week 12	3.2 ± 0.6	2.5 ± 0.2	1.7 ± 0.2	1.8 ± 0.1
**Urine phosphate excretion**				
(mg/24 h)	33.1 ± 1	23.6 ± 3.6	82.8 ± 29.9	59.8 ± 7.5

BUN = blood urea nitrogen; K_2_ = MK-7.

### 3.2. Cardiovascular Calcification

We directly assessed cardiovascular calcification by histology (von Kossa) and chemical analysis (AAS = atomic absorption spectroscopy) in the aorta, myocardium, and kidneys in the various treatment groups. AAS is a sensitive method for detecting total tissue calcium amount [37]. In the aortas we performed additional immunohistochemistry for alkaline phosphatase to evaluate the downstream effects of mineralization in terms of VSMC dedifferentiation.

**Figure 2:**
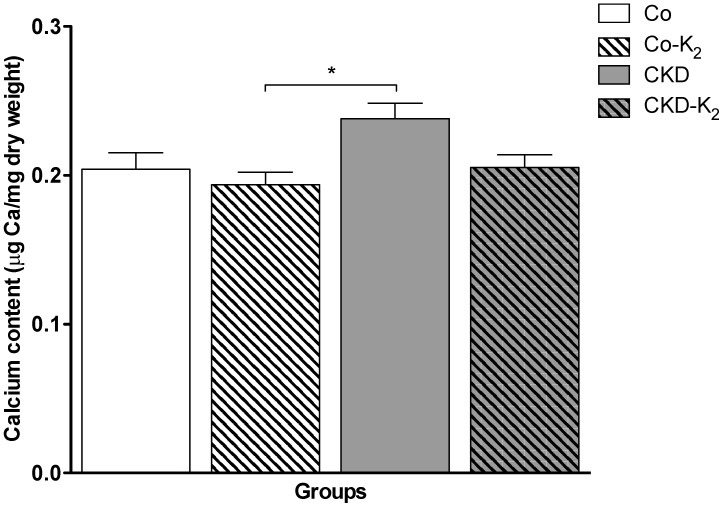
Aortic calcium content. Measurement of aortic calcium content was performed by atomic absorption spectrometry and expressed as μg Ca/mg dry weight tissue. CKD animals display a significant increase in aortic calcium content compared to Co-K_2_ animals. Co = control group; Co-K_2_ = MK-7 supplemented control group; CKD = 5/6 nephrectomized group; CKD-K_2_ = MK-7 supplemented 5/6 nephrectomized group; mg = milligrams; µg = micrograms. Significant differences: * *p* < 0.05.

**Figure 3:**
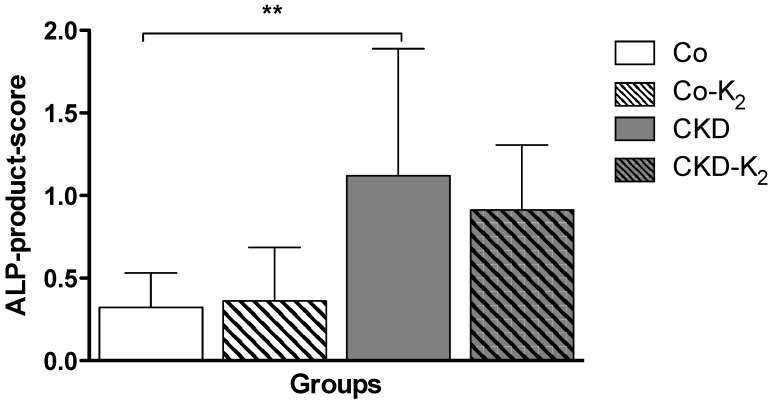
Alkaline phosphatase (ALP) in aortic tissues. ALP was measured to detect local vascular osteochondrogenic activity. Positive staining for ALP is expressed as product score: ALP-positive staining areal × intensity. ALP-product-score is significantly increased in CKD animals as compared to Co animals. Co = control group; Co-K_2_ = MK-7 supplemented control group; CKD = 5/6 nephrectomized group; CKD-K_2_ = MK-7 supplemented 5/6 nephrectomized group. Significant differences: ** *p* < 0.01.

**Aorta:** While von Kossa staining did not encounter calcification in any treatment group, we detected significant differences in the chemical calcium analysis applying AAS. Animals from the CKD group displayed an induction of aortic calcium content compared to controls by about 30% (*p* < 0.05). MK-7 supplementation abolished aortic calcification, thus CKD-K_2_ rats displayed aortic calcium content indistinguishable from controls (Figure 2). In line with the calcium accumulation in the CKD group, we detected an intensified ALP staining in aortic tissue samples of CKD animals compared to control groups (CKD *vs.* Co, *p* < 0.01) and CKD-K_2_ animals (Figure 3).

**Figure 4:**
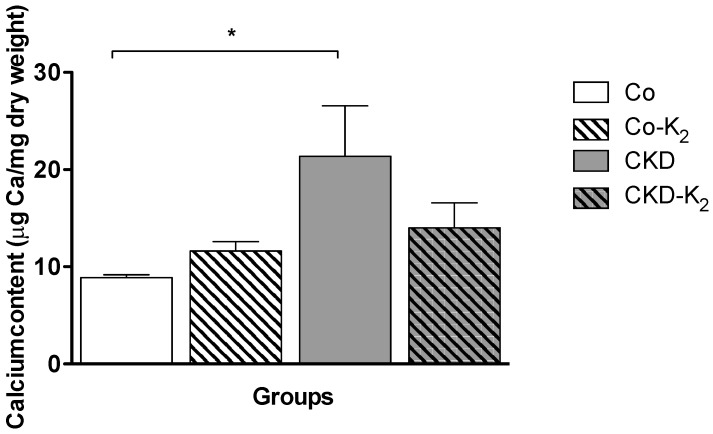
Myocardial calcium content. Measurement of myocardial calcium content using atomic absorption spectrometry in μg Ca/mg dry weight tissue. CKD animals display a significantly increased myocardial calcium content compared to Co animals. Co = control group; Co-K_2_ = MK-7 supplemented control group; CKD = 5/6 nephrectomized group; CKD-K_2_ = MK-7 supplemented 5/6 nephrectomized group; mg = milligrams; µg = micrograms. Significant differences: * *p* < 0.05.

**Myocardium:** Similar to our observations in aortic tissue, we did not detect overt myocardial calcification by von Kossa staining in any treatment group. Applying chemical analysis by AAS, myocardial tissues of the CKD group displayed a 2.4-fold increased calcium concentration compared to controls (CKD *vs.* Co, *p* < 0.05). Again, MK-7 supplementation inhibited calcium accumulation in the CKD-K_2_ rats with calcium tissue concentrations in the range of control rats (Figure 4).

**Figure 5:**
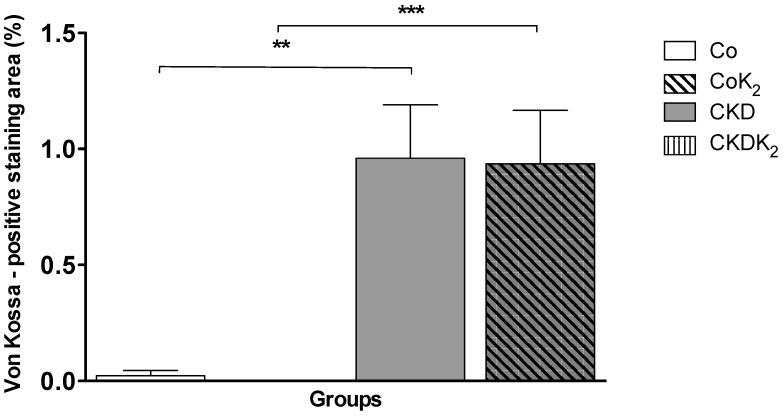
Renal calcification. Von Kossa positive staining area expressed as percentage of tissue area. In 5/6 nephrectomized animals significantly more von Kossa positivity was measured as compared to the control groups. No differences were seen between CKD and CKD-K_2_ treated groups. Co = control group; Co-K_2_ = MK-7 supplemented control group; CKD = 5/6 nephrectomized group; CKD-K_2_ = MK-7 supplemented 5/6 nephrectomized group. Significant differences: ** *p* < 0.01, *** *p* < 0.001.

**Figure 6:**
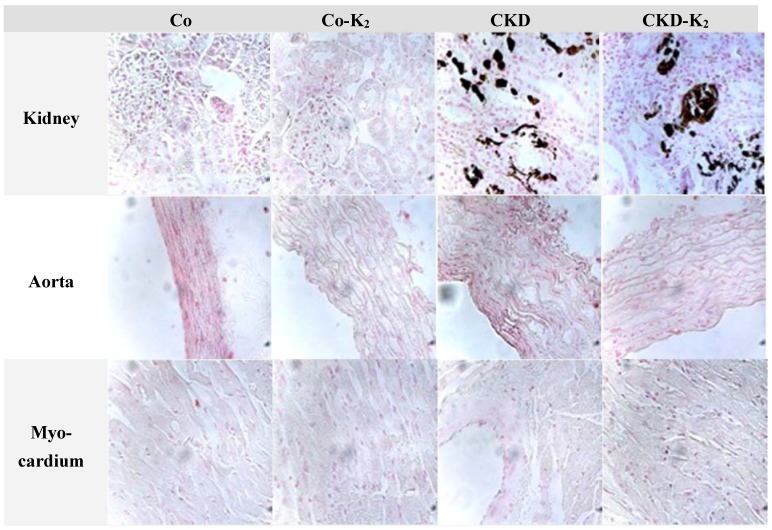
Representative sections of von Kossa stained tissue samples. Microscopically visible von Kossa positive staining was only detected in kidney tissue samples of CKD and CDK-K_2_ animals. Magnification 40×; Counterstain: nuclear fast red; Co = control group; Co-K_2_ = MK-7 supplemented control group; CKD = 5/6 nephrectomized group; CKD-K_2_ = MK-7 supplemented 5/6 nephrectomized group.

**Kidney**: In renal tissues of CKD and CKD-K_2_ animals we detected tubular calcification easily visible on von Kossa histology (Figure 5 and Figure 6). Quantitative analysis detected no effect of MK-7 supplementation on renal calcification.

### 3.3. Structural Alterations in Experimental CKD

**Figure 7:**
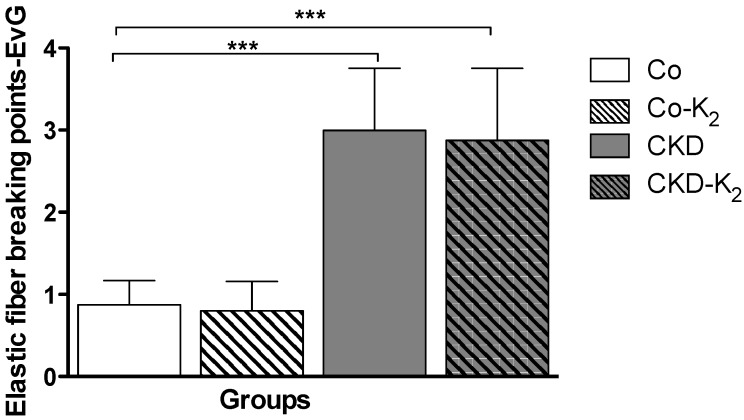
Elastic fiber breaking points. Elastica van Gieson (EvG) staining of aortic tissue samples with focus on elastic fiber breaking points. CKD animals display significantly more elastic fiber breaking points compared to Co animals. Co = control group; Co-K_2_ = MK-7 supplemented control group; CKD = 5/6 nephrectomized group; CKD-K_2_ = MK-7 supplemented 5/6 nephrectomized group. Significant differences: *** *p* < 0.001.

**Fiber breaking points:** In order to evaluate the structural alterations induced by 5/6 nephrectomy and a high phosphate diet, we performed Elastica van Gieson staining of the aorta to illustrate elastic fiber breaking points. Both interventional groups (CKD, CKD-K_2_) displayed significantly more elastic fiber breaking points compared to the control groups (CKD *vs.* Co, *p* < 0.001) (Figure 7). MK-7 supplementation had no effect on fiber breaking points in controls or CKD rats, respectively.

**Figure 8:**
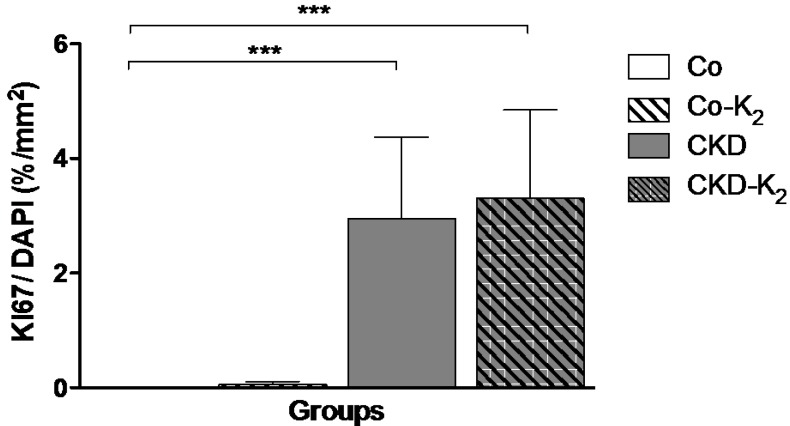
Proliferative response. Ratio of positive staining area for Ki67 per DAPI positive staining area in % per mm^2^. DAPI values do not differ significantly between groups. CKD animals display significantly more Ki67 positive staining area per DAPI positive staining area compared to Co animals. Co = control group; Co-K_2_ = MK-7 supplemented control group; CKD = 5/6 nephrectomized group; CKD-K_2_ = MK-7 supplemented 5/6 nephrectomized group; mm = millimeters. Significant differences: *** *p* < 0.001.

**Proliferative response:** Immunofluorescence staining of Ki67/DAPI revealed markedly increased cell proliferation in CKD and CKD-K_2_ animals compared to controls (CKD *vs.* Co, *p* < 0.001). MK-7 supplementation did not alter the proliferative response to the CKD stimulus (Figure 8).

### 3.4. Cardiovascular Function

**Figure 9:**
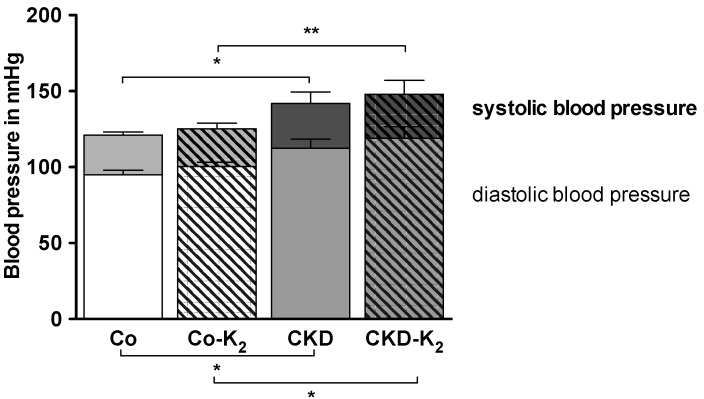
Systolic and diastolic blood pressure. Systolic and diastolic blood pressure after 12 weeks of treatment. CKD animals developed significantly increased systolic and diastolic blood pressure values compared to controls. Co = control group; Co-K_2_ = MK-7 supplemented control group; CKD = 5/6 nephrectomized group; CKD-K_2_ = MK-7 supplemented 5/6 nephrectomized group; mmHg = millimeters of mercury. Significant differences: * *p* < 0.05, ** *p* < 0.01.

**Blood pressure:** CKD and CKD-K_2_ animals developed arterial hypertension (Figure 9). After 12 weeks, systolic as well as diastolic blood pressure values were elevated by 15–20 mmHg in CKD rats compared to controls (CKD-K_2_ *vs.* Co-K_2_, *p* < 0.01). MK-7 supplementation did not affect blood pressure (Figure 9).

**Figure 10:**
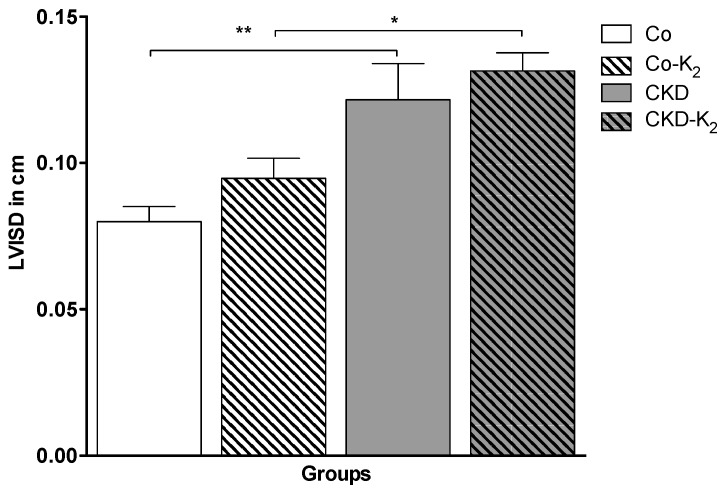
Echocardiography of diastolic interventricular septum diameter (LVISD) in cm. Echocardiography was performed in all animals after 12 weeks of study protocol. CKD animals developed significantly increased LVISD compared to controls. Co = control group; Co-K_2_ = MK-7 supplemented control group; CKD = 5/6 nephrectomized group; CKD-K_2_ = MK-7 supplemented 5/6 nephrectomized group; cm = centimeters. Significant differences: * *p* < 0.05, ** *p* < 0.01.

**Echocardiography:** To quantify the impact of CKD and arterial hypertension on cardiovascular function we analyzed hypertrophy, contractility, and valvular function in the various treatment groups. CKD and CKD-K_2_ animals exhibited significant myocardial hypertrophy, as depicted by increased diameters of the interventricular septum (by about 50%; CKD *vs.* Co, *p* < 0.01) (Figure 10). Myocardial contractility, as detected by fractional shortening, remained normal in all the animals studied. No relevant valvular disease or overt signs of valvular calcification were detected throughout the experiments (data not shown).

### 3.5. Aortic mRNA Expression

**Figure 11:**
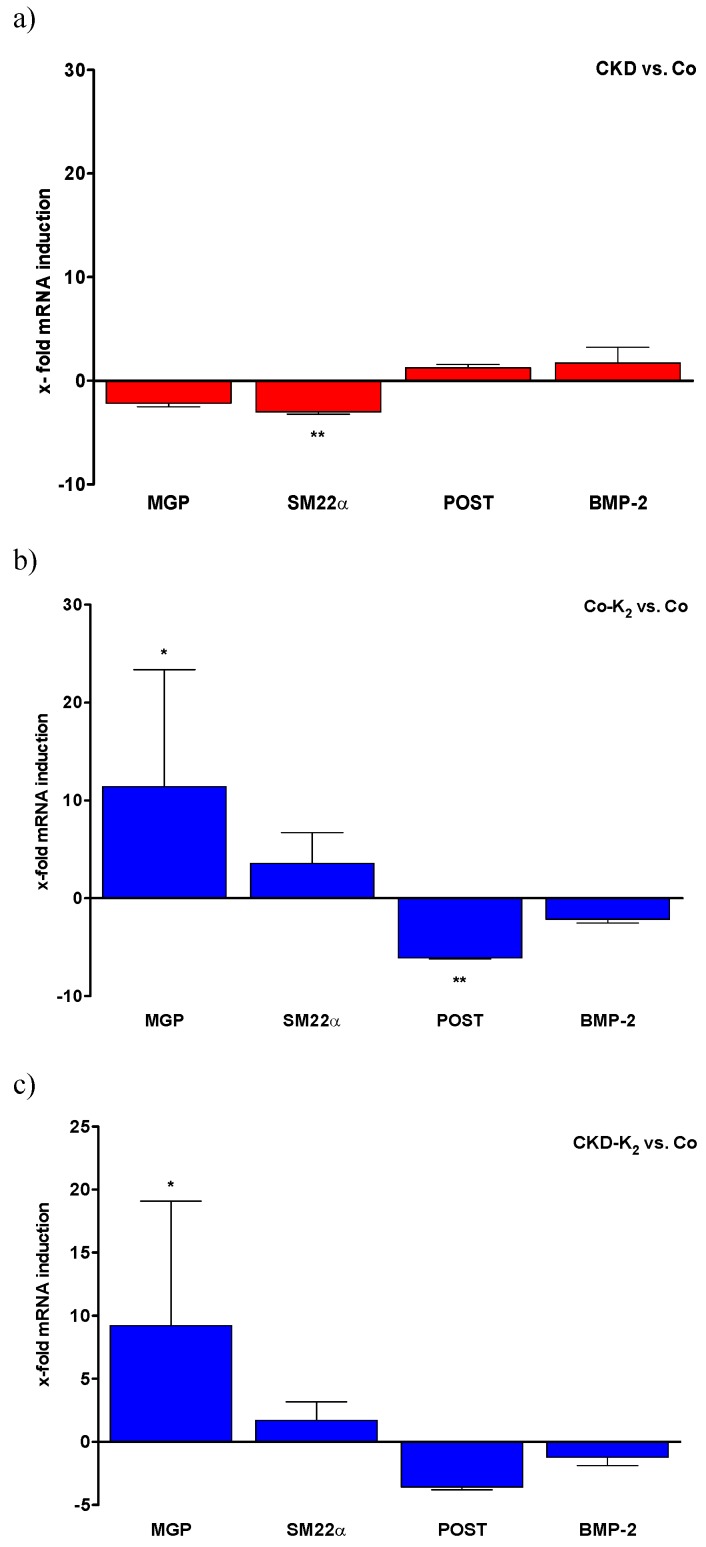
Aortic gene expression. The effect of CKD and MK-7 supplementation on aortic gene expression of calcification modifiers as x-fold induction compared to control group. Red columns depict pro-calcific changes, while alterations associated with potential calcification inhibition are assigned with blue columns. (**a**) In CKD animals SM22α expression is significantly decreased, whereas MGP expression is non-significantly decreased as compared to Co animals. (**b**) In Co-K_2_ animals MGP mRNA concentration is significantly increased, whereas POST mRNA concentration is significantly decreased compared to Co animals. (**c**) In CKD-K_2_ animals MGP mRNA concentration is significantly and SM22α mRNA concentration is tendentially increased compared to Co animals. Co = control group; Co-K_2_ = MK-7 supplemented control group; CKD = 5/6 nephrectomized group; CKD-K_2_ = MK-7 supplemented 5/6 nephrectomized group; MGP = matrix Gla-protein; POST = periostin; BMP-2 = bone morphogenetic protein 2. Significant differences: * *p* < 0.05, ** *p* < 0.01.

We analyzed regulation of relevant activators and inhibitors of VC in aortic tissue samples by quantitative real-time PCR. In a stepwise analysis, we investigated separately the effect of CKD (Figure 11a), MK-7 supplementation (Figure 11b), and finally the effect of MK-7 supplementation in CKD rats (Figure 11c), each compared to controls. For more clarity, pro-calcific changes are depicted by red columns while alterations associated with potential calcification inhibition are assigned with blue columns.

**CKD compared to controls:** The induction of CKD was associated with pro-calcific mRNA alterations within the aortas of the animals. MGP and SM22α gene expression was markedly decreased in CKD animals, while periostin and BMP-2 gene expression was increased compared to controls (Figure 11a).

**MK-7 supplementation compared to controls:** MK-7 supplementation revealed a significant induction of aortic MGP mRNA while procalcifing periostin mRNA levels were found to be significantly reduced (Figure 11b).

**MK-7 supplementation and CKD compared to controls:** MK-7 supplementation in CKD was associated with a calcification inhibitory effect in the aortic mRNA repertoire. Of note, we detected a significant induction of MGP mRNA levels. Moreover, the CKD-mediated reduction of SM22α mRNA was abolished, as depicted by a trend towards increased SM22α mRNA (Figure 11c).

## 4. Discussion

In this study we demonstrate that high-dose MK-7 supplementation inhibits calcification in a murine model of CKD-induced cardiovascular calcification. The effect of MK-7 is—at least in part—mediated via MGP and subsequent inhibition of ectopic calcification.

### 4.1. CKD and High Phosphate Diet

In our study we used a murine model of 5/6 nephrectomy and high phosphate diet to induce CKD-related cardiovascular damage. Shobeiri *et al.* compared different experimental CKD models for the induction of VC [38]. They observed a 2.2-fold increased serum creatinine concentration in 5/6 nephrectomized rats eight weeks after surgery, with corresponding values for BUN and creatinine clearance [38]. Our findings are similar with respect to serum creatinine and BUN levels and creatinine clearance but differ in severity of kidney injury. We observed moderate kidney injury, whereas others report severe kidney injury after 5/6 nephrectomy. This difference might arise from the technique we used to perform the 5/6 nephrectomy. We used unilateral nephrectomy, followed by ligation of the poles of the remaining kidney, whereas most studies describe surgical resection of the kidney poles or embolization of the pole-supplying arteries to induce renal impairment [38,39]. As expected, animals from CKD and CKD-K_2_ groups gained less weight as compared to control animals, which is in accordance with previous studies [39]. Moreover, CKD and CKD-K_2_ animals did not developed anemia. This finding is consistent with moderate kidney injury, since anemia is most frequently seen in patients with severe renal impairment [40].

### 4.2. Cardiovascular Calcification

Animals in the CKD groups exhibited modest VCs with concomitant alkaline phosphatase presence. ALP may serve as a marker for osteoblastic activity [41]. However, increased deposits of ALP in the vasculature of animals in the CKD groups are also associated with the transdifferentiation of VSMCs towards osteochondrogenic-like cells [42]. These data are supported by the significantly decreased expression of SM22α, a protein specific for VSMCs. Our data are in line with previous observations that SM22α mRNA levels are decreased in a murine model of VC [43,44]. Moreover, we detected increased expression levels of periostin and BMP-2. Periostin is associated with increased extracellular matrix turnover, as seen in diseases like CKD [45,46]. Additionally, periostin is inversely correlated with decreased kidney function [47], which is in line with our results. BMP-2 expression is increased in atherosclerotic plaques [48] and in *in vitro* models of VC [49], supporting our data of CKD-induced VC.

MGP is a vitamin K-dependent protein known to inhibit VC. Functionality of MGP is dependent on vitamin K. We demonstrate that MGP expression is decreased in CKD animals as compared to controls. It has been described that MGP synthesis increases in response to VC [18,49]. Diabetes and CKD have an inhibitory effect on the protective upregulation of MGP expression, which is a possible pathomechanism for CKD-induced VC [50].

For the first time, we demonstrate that vitamin K (MK-7) affects MGP mRNA expression (>10-fold induction in controls and CKD rats) within the vascular wall. Earlier studies [17] showed that only locally expressed MGP in the arterial wall—and not systemic MGP over-expression—inhibits VC. Our results point to protective effects of MK-7 supplementation against imminent VC at the transcriptional level. There is evidence that MK-4 mediates MGP mRNA expression via activation of the SXR (steroid and xenobiotic receptor) nuclear receptor [51]. MK-4 has been shown to activate SXR in a dose-dependent manner [51]. Further studies are required to examine whether this observation can be replicated by MK-7. It is well known that vitamin K is a cofactor for the posttranslational carboxylation of MGP [16]. In our study we did not measure uncarboxylated and carboxylated MGP status.

MK-7 supplementation increased SM22α expression, whereas periostin and BMP-2 expression decreased, indicating that MK-7 affects VSMC phenotypic switching. This corroborates recent findings that the vitamin K-dependent Gla-rich protein (GRP) affects VC via VSMC phenotype modulation [52]. Thus, the impact of CKD on cardiovascular calcification might be counteracted by MK-7 supplementation via regulation of MGP expression and activity, and VSMC phenotypic switching.

Animals in both CKD and CKD-K_2_ (MK-7 supplemented) groups significantly developed renal calcium deposits, particularly in the tubules. The significant amount of calcium deposits may be explained by the increased phosphate clearance by the remaining nephrons in combination with the high phosphate diet [53]. Phosphate levels in CKD play an important role in the development of cardiovascular calcification [54,55].

Interestingly, MK-7 supplementation normalized CKD-induced increased serum phosphate concentrations. More importantly, MK-7 supplementation inhibited both vascular and myocardial calcification in CKD animals on a high phosphate diet. Our results demonstrate a protective effect of MK-7 supplementation on early stages of cardiovascular calcification. Our results support previous data from our group, showing that both VK_1_ and MK-4 supplementation reverses vitamin K antagonist-induced VC in rats [34]. To our best knowledge, this is the first study demonstrating a protective effect of MK-7 supplementation in a murine model of 5/6 nephrectomy-induced CKD. Compared to commonly applied models to induce VC (adenine diet, gene knockout animals, high-dose warfarin treatment), the VC calcification model applied in the current manuscript with mild CKD and phosphate loading more closely resembles the clinical situation of early CKD. As a matter of fact, cardiovascular mortality is already profoundly increased at early stages of CKD (e.g., below 60 mL/min GFR), rendering this model an attractive option to study the pathophysiological processes of imminent calcification and potential treatment options related to early CKD. Our results support the rationale for prophylactic supplementation of MK-7 in patients at risk for development of cardiovascular calcification. It has been shown that MK-7 supplementation is safe and has no side effects, both in experimental animals and humans [31,32,56]. Patients with CKD have been shown to be subclinically vitamin K deficient [21,22,57]. In an observational study in hemodialysis patients it was reported that a low circulating concentration of MK-4 was a predictor of aortic calcification [58], but this claim remains to be confirmed or refuted. Our group showed that MK-7 supplementation reduces levels of inactive MGP, osteocalcin, and PIVKA-II in hemodialysis patients [27]. It is tempting to speculate whether these results can be transferred to patients, a hypothesis that is currently being investigated in several studies [59].

### 4.3. Structural and Functional Alterations

In our model, CKD and a high phosphate diet induced increased systolic and diastolic blood pressure with cardiac hypertrophy. It is known that CKD and VC are associated with arterial hypertension [54] and reduced vascular compliance, with subsequent increased systolic and decreased diastolic blood pressure values [60]. Hypertension is most likely a consequence of kidney disease since blood pressure values were similarly elevated in both CKD and CKD-K_2_ animals. CKD and hypertension are associated with vascular remodeling and increased cell proliferation [61,62]. VSMC proliferation increased in CKD and CKD-K_2_ groups but was not affected by MK-7 treatment. Additionally, we detected increased elastic fiber breaking points in the tunica media, indicative of loss of vascular integrity in animals of the CKD and CKD-K_2_ groups [63].

Importantly, we observed an inhibition of calcification of damaged tissues by MK-7 supplementation, whereas MK-7 was not able to continuously block the overwhelming harmful milieu of CKD and a high phosphate diet.

## 5. Conclusions

Our data provide evidence for a protective effect of high-dose MK-7 supplementation on cardiovascular calcification. MK-7 supplementation could not prevent CKD-associated hypertension and hypertrophy but prevented calcification of affected tissues. Since vitamin K has no reported side effects, it seems a promising therapeutic agent [59]. The effect of MK-7 is likely to act via vitamin K-dependent proteins such as MGP and it is tempting to speculate whether these results can be transferred to CKD patients.

## References

[1] Allison M.A., Criqui M.H., Wright C.M. (2004). Patterns and risk factors for systemic calcified atherosclerosis. Arterioscler. Thromb. Vasc. Biol..

[2] Giachelli C.M. (2009). The emerging role of phosphate in vascular calcification. Kidney Int..

[3] Schurgin S., Rich S., Mazzone T. (2001). Increased prevalence of significant coronary artery calcification in patients with diabetes. Diabetes Care.

[4] Vliegenthart R., Oudkerk M., Hofman A., Oei H.H., van Dijck W., van Rooij F.J., Witteman J.C. (2005). Coronary calcification improves cardiovascular risk prediction in the elderly. Circulation.

[5] Ketteler M., Schlieper G., Floege J. (2006). Calcification and cardiovascular health—New insights into an old phenomenon. Hypertension.

[6] Wu M., Rementer C., Giachelli C.M. (2013). Vascular calcification: An update on mechanisms and challenges in treatment. Calcif. Tissue Int..

[7] Moe S.M., O’Neill K.D., Duan D., Ahmed S., Chen N.X., Leapman S.B., Fineberg N., Kopecky K. (2002). Medial artery calcification in esrd patients is associated with deposition of bone matrix proteins. Kidney Int..

[8] Demer L.L., Tintut Y. (2008). Vascular calcification: Pathobiology of a multifaceted disease. Circulation.

[9] Chatrou M.L., Winckers K., Hackeng T.M., Reutelingsperger C.P., Schurgers L.J. (2012). Vascular calcification: The price to pay for anticoagulation therapy with vitamin K-antagonists. Blood Rev..

[10] Reaven P.D., Sacks J. (2005). Coronary artery and abdominal aortic calcification are associated with cardiovascular disease in type 2 diabetes. Diabetologia.

[11] Okuno S., Ishimura E., Kitatani K., Fujino Y., Kohno K., Maeno Y., Maekawa K., Yamakawa T., Imanishi Y., Inaba M. (2007). Presence of abdominal aortic calcification is significantly associated with all-cause and cardiovascular mortality in maintenance hemodialysis. Am. J. Kidney Dis..

[12] Schurgers L.J., Cranenburg E.C.M., Vermeer C. (2008). Matrix gla-protein: The calcification inhibitor in need of vitamin k. Thromb. Haemost..

[13] Price P.A., Urist M.R., Otawara Y. (1983). Matrix gla protein, a new gamma-carboxyglutamic acid-containing protein which is associated with the organic matrix of bone. Biochem. Biophys. Res. Commun..

[14] Luo G., Ducy P., McKee M.D., Pinero G.J., Loyer E., Behringer R.R., Karsenty G. (1997). Spontaneous calcification of arteries and cartilage in mice lacking matrix gla protein. Nature.

[15] Munroe P.B., Olgunturk R.O., Fryns J.P., van Maldergem L., Ziereisen F., Yuksel B., Gardiner R.M., Chung E. (1999). Mutations in the gene encoding the human matrix gla protein cause keutel syndrome. Nat. Genet..

[16] Schurgers L.J., Uitto J., Reutelingsperger C.P. (2013). Vitamin k-dependent carboxylation of matrix gla-protein: A crucial switch to control ectopic mineralization. Trends Mol. Med..

[17] Murshed M., Schinke T., McKee M.D., Karsenty G. (2004). Extracellular matrix mineralization is regulated locally; different roles of two gla-containing proteins. J. Cell Biol..

[18] Schurgers L.J., Teunissen K.J., Knapen M.H., Kwaijtaal M., van Diest R., Appels A., Reutelingsperger C.P., Cleutjens J.P., Vermeer C. (2005). Novel conformation-specific antibodies against matrix gamma-carboxyglutamic acid (gla) protein: Undercarboxylated matrix gla protein as marker for vascular calcification. Arterioscler. Thromb. Vasc. Biol..

[19] Berkner K.L., Runge K.W. (2004). The physiology of vitamin k nutriture and vitamin k-dependent protein function in atherosclerosis. J. Thromb. Haemost..

[20] Rennenberg R.J.M.W., van Varik B.J., Schurgers L.J., Hamulyak K., ten Cate H., Leiner T., Vermeer C., de Leeuw P.W., Kroon A.A. (2010). Chronic coumarin treatment is associated with increased extracoronary arterial calcification in humans. Blood.

[21] Pilkey R.M., Morton A.R., Boffa M.B., Noordhof C., Day A.G., Su Y.H., Miller L.M., Koschinsky M.L., Booth S.L. (2007). Subclinical vitamin k deficiency in hemodialysis patients. Am. J. Kidney Dis..

[22] Schurgers L.J., Barreto D.V., Barreto F.C., Liabeuf S., Renard C., Magdeleyns E.J., Vermeer C., Choukroun G., Massy Z.A. (2010). The circulating inactive form of matrix gla protein is a surrogate marker for vascular calcification in chronic kidney disease: A preliminary report. Clin. J. Am. Soc. Nephrol..

[23] Shea M.K., O’Donnell C.J., Vermeer C., Magdeleyns E.J.P., Crosier M.D., Gundberg C.M., Ordovas J.M., Kritchevsky S.B., Booth S.L. (2011). Circulating uncarboxylated matrix gla protein is associated with vitamin k nutritional status, but not coronary artery calcium, in older adults. J. Nutr..

[24] Ueland T., Gullestad L., Dahl C.P., Aukrust P., Aakhus S., Solberg O.G., Vermeer C., Schurgers L.J. (2010). Undercarboxylated matrix gla protein is associated with indices of heart failure and mortality in symptomatic aortic stenosis. J. Intern. Med..

[25] Ueland T., Dahl C.P., Gullestad L., Aakhus S., Broch K., Skardal R., Vermeer C., Aukrust P., Schurgers L.J. (2011). Circulating levels of non-phosphorylated undercarboxylated matrix gla protein are associated with disease severity in patients with chronic heart failure. Clin. Sci..

[26] Cranenburg E.C., Koos R., Schurgers L.J., Magdeleyns E.J., Schoonbrood T.H., Landewe R.B., Brandenburg V.M., Bekers O., Vermeer C. (2010). Characterisation and potential diagnostic value of circulating matrix gla protein (mgp) species. Thromb. Haemost..

[27] Westenfeld R., Krueger T., Schlieper G., Cranenburg E.C., Magdeleyns E.J., Heidenreich S., Holzmann S., Vermeer C., Jahnen-Dechent W., Ketteler M. (2012). Effect of vitamin k2 supplementation on functional vitamin k deficiency in hemodialysis patients: A randomized trial. Am. J. Kidney Dis..

[28] Gonnet M., Lethuaut L., Boury F. (2010). New trends in encapsulation of liposoluble vitamins. J. Control. Release.

[29] Gijsbers B.L., Jie K.S., Vermeer C. (1996). Effect of food composition on vitamin k absorption in human volunteers. Br. J. Nutr..

[30] Schurgers L.J., Vermeer C. (2000). Determination of phylloquinone and menaquinones in food. Effect of food matrix on circulating vitamin k concentrations. Haemostasis.

[31] Beulens J.W.J., Bots M.L., Atsma F., Bartelink M.L.E.L., Prokop M., Geleijnse J.M., Witteman J.C.M., Grobbee D.E., van der Schouw Y.T. (2009). High dietary menaquinone intake is associated with reduced coronary calcification. Atherosclerosis.

[32] Geleijnse J.M., Vermeer C., Grobbee D.E., Schurgers L.J., Knapen M.H., van der Meer I.M., Hofman A., Witteman J.C. (2004). Dietary intake of menaquinone is associated with a reduced risk of coronary heart disease: The rotterdam study. J. Nutr..

[33] Kruger T., Oelenberg S., Kaesler N., Schurgers L.J., van de Sandt A.M., Boor P., Schlieper G., Brandenburg V.M., Fekete B.C., Veulemans V. (2013). Warfarin induces cardiovascular damage in mice. Arterioscler. Thromb. Vasc. Biol..

[34] Schurgers L.J., Spronk H.M., Soute B.A., Schiffers P.M., DeMey J.G., Vermeer C. (2007). Regression of warfarin-induced medial elastocalcinosis by high intake of vitamin k in rats. Blood.

[35] Spronk H.M., Soute B.A., Schurgers L.J., Thijssen H.H., de Mey J.G., Vermeer C. (2003). Tissue-specific utilization of menaquinone-4 results in the prevention of arterial calcification in warfarin-treated rats. J. Vasc. Res..

[36] Perez-Ruiz L., Ros-Lopez S., Cardus A., Fernandez E., Valdivielso J.M. (2006). A forgotten method to induce experimental chronic renal failure in the rat by ligation of the renal parenchyma. Nephron. Exp. Nephrol..

[37] Ng K., Hildreth C.M., Phillips J.K., Avolio A.P. (2011). Aortic stiffness is associated with vascular calcification and remodeling in a chronic kidney disease rat model. Am. J. Physiol. Renal Physiol..

[38] Shobeiri N., Adams M.A., Holden R.M. (2010). Vascular calcification in animal models of ckd: A review. Am. J. Nephrol..

[39] Fleck C., Appenroth D., Jonas P., Koch M., Kundt G., Nizze H., Stein G. (2006). Suitability of 5/6 nephrectomy (5/6nx) for the induction of interstitial renal fibrosis in rats—Influence of sex, strain, and surgical procedure. Exp. Toxicol. Pathol..

[40] El-Achkar T.M., Ohmit S.E., McCullough P.A., Crook E.D., Brown W.W., Grimm R., Bakris G.L., Keane W.F., Flack J.M. (2005). Higher prevalence of anemia with diabetes mellitus in moderate kidney insufficiency: The kidney early evaluation program. Kidney Int..

[41] Shioi A., Katagi M., Okuno Y., Mori K., Jono S., Koyama H., Nishizawa Y. (2002). Induction of bone-type alkaline phosphatase in human vascular smooth muscle cells: Roles of tumor necrosis factor-alpha and oncostatin m derived from macrophages. Circ. Res..

[42] Kendrick J., Chonchol M. (2011). The role of phosphorus in the development and progression of vascular calcification. Am. J. Kidney Dis..

[43] El-Abbadi M.M., Pai A.S., Leaf E.M., Yang H.Y., Bartley B.A., Quan K.K., Ingalls C.M., Liao H.W., Giachelli C.M. (2009). Phosphate feeding induces arterial medial calcification in uremic mice: Role of serum phosphorus, fibroblast growth factor-23, and osteopontin. Kidney Int..

[44] Steitz S.A., Speer M.Y., Curinga G., Yang H.Y., Haynes P., Aebersold R., Schinke T., Karsenty G., Giachelli C.M. (2001). Smooth muscle cell phenotypic transition associated with calcification: Upregulation of cbfa1 and downregulation of smooth muscle lineage markers. Circ. Res..

[45] Hakuno D., Kimura N., Yoshioka M., Mukai M., Kimura T., Okada Y., Yozu R., Shukunami C., Hiraki Y., Kudo A. (2010). Periostin advances atherosclerotic and rheumatic cardiac valve degeneration by inducing angiogenesis and mmp production in humans and rodents. J. Clin. Invest..

[46] Hixson J.E., Shimmin L.C., Montasser M.E., Kim D.K., Zhong Y., Ibarguen H., Follis J., Malcom G., Strong J., Howard T. (2011). Common variants in the periostin gene influence development of atherosclerosis in young persons. Arterioscler. Thromb. Vasc. Biol..

[47] Sen K., Lindenmeyer M.T., Gaspert A., Eichinger F., Neusser M.A., Kretzler M., Segerer S., Cohen C.D. (2011). Periostin is induced in glomerular injury and expressed de novo in interstitial renal fibrosis. Am. J. Pathol..

[48] Bostrom K., Watson K.E., Horn S., Wortham C., Herman I.M., Demer L.L. (1993). Bone morphogenetic protein expression in human atherosclerotic lesions. J. Clin. Invest..

[49] Ciceri P., Elli F., Brenna I., Volpi E., Romagnoli S., Tosi D., Braidotti P., Brancaccio D., Cozzolino M. (2013). Lanthanum prevents high phosphate-induced vascular calcification by preserving vascular smooth muscle lineage markers. Calcif. Tissue Int..

[50] Kaesler N., Magdeleyns E., Herfs M., Schettgen T., Brandenburg V., Fliser D., Vermeer C., Floege J., Schlieper G., Kruger T. (2014). Impaired vitamin k recycling in uremia is rescued by vitamin k supplementation. Kidney Int..

[51] Tabb M.M., Sun A., Zhou C., Grun F., Errandi J., Romero K., Pham H., Inoue S., Mallick S., Lin M. (2003). Vitamin k2 regulation of bone homeostasis is mediated by the steroid and xenobiotic receptor sxr. J. Biol. Chem..

[52] Viegas C.S., Rafael M.S., Enriquez J.L., Teixeira A., Vitorino R., Luis I.M., Costa R.M., Santos S., Cavaco S., Neves J. (2015). Gla-rich protein acts as a calcification inhibitor in the human cardiovascular system. Arterioscler. Thromb. Vasc. Biol..

[53] Tsuchiya N., Matsushima S., Takasu N., Kyokawa Y., Torii M. (2004). Glomerular calcification induced by bolus injection with dibasic sodium phosphate solution in sprague-dawley rats. Toxicol. Pathol..

[54] Eknoyan G., Levin N.W. (2002). K/doqi clinical practice guidelines for chronic kidney disease: Evaluation, classification, and stratification. Am. J. Kidney Dis..

[55] McCarty M.F., DiNicolantonio J.J. (2014). Bioavailable dietary phosphate, a mediator of cardiovascular disease, may be decreased with plant-based diets, phosphate binders, niacin, and avoidance of phosphate additives. Nutrition.

[56] Pucaj K., Rasmussen H., Moller M., Preston T. (2011). Safety and toxicological evaluation of a synthetic vitamin k2, menaquinone-7. Toxicol. Mech. Methods.

[57] Holden R.M., Morton A.R., Garland J.S., Pavlov A., Day A.G., Booth S.L. (2010). Vitamins k and d status in stages 3–5 chronic kidney disease. Clin. J. Am. Soc. Nephrol..

[58] Fusaro M., Noale M., Viola V., Galli F., Tripepi G., Vajente N., Plebani M., Zaninotto M., Guglielmi G., Miotto D. (2012). Vitamin k, vertebral fractures, vascular calcifications, and mortality: Vitamin k italian (viki) dialysis study. J. Bone Miner. Res..

[59] Brandenburg V.M., Schurgers L.J., Kaesler N., Pusche K., van Gorp R.H., Leftheriotis G., Reinartz S., Koos R., Kruger T. (2015). Prevention of vasculopathy by vitamin k supplementation: Can we turn fiction into fact?. Atherosclerosis.

[60] Taddei S., Nami R., Bruno R.M., Quatrini I., Nuti R. (2011). Hypertension, left ventricular hypertrophy and chronic kidney disease. Heart Fail. Rev..

[61] Feihl F., Liaudet L., Levy B.I., Waeber B. (2008). Hypertension and microvascular remodelling. Cardiovasc. Res..

[62] Li S., Wang X., Li Y., Kost C.K., Martin D.S. (2013). Bortezomib, a proteasome inhibitor, attenuates angiotensin ii-induced hypertension and aortic remodeling in rats. PLoS One.

[63] Ro A., Kageyama N. (2013). Pathomorphometry of ruptured intracranial vertebral arterial dissection: Adventitial rupture, dilated lesion, intimal tear, and medial defect. J. Neurosurg..

